# Supplementary figures and images for: Targeting the tsetse-trypanosome interplay using genetically engineered Sodalis glossinidius
Source: PLoS Pathog. 2022 Mar 10;18(3):e1010376. doi: 10.1371/journal.ppat.1010376 (PMC8939806; doi:10.1371/journal.ppat.1010376)

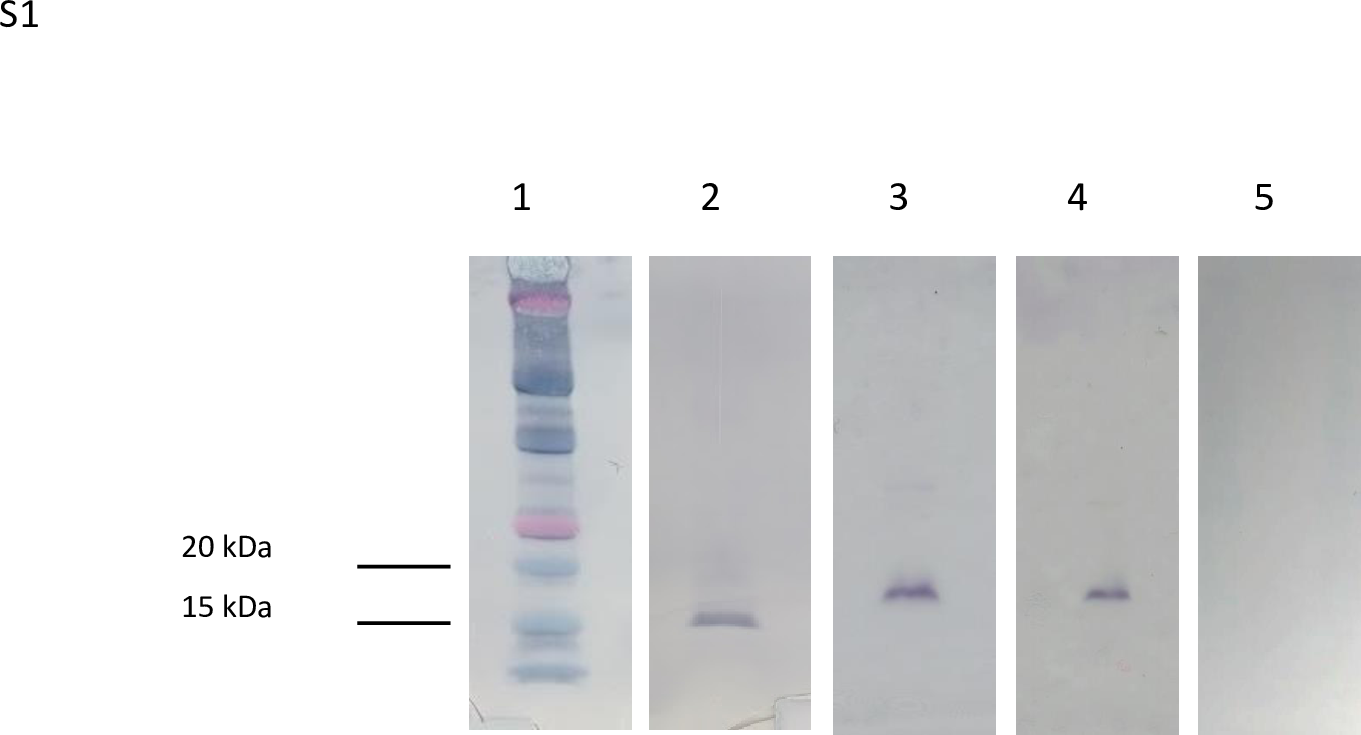

Supplement: S1 Fig — Concentrated spent medium was tested using an anti-His antibody. (TIF) [file ppat.1010376.s001.tif]

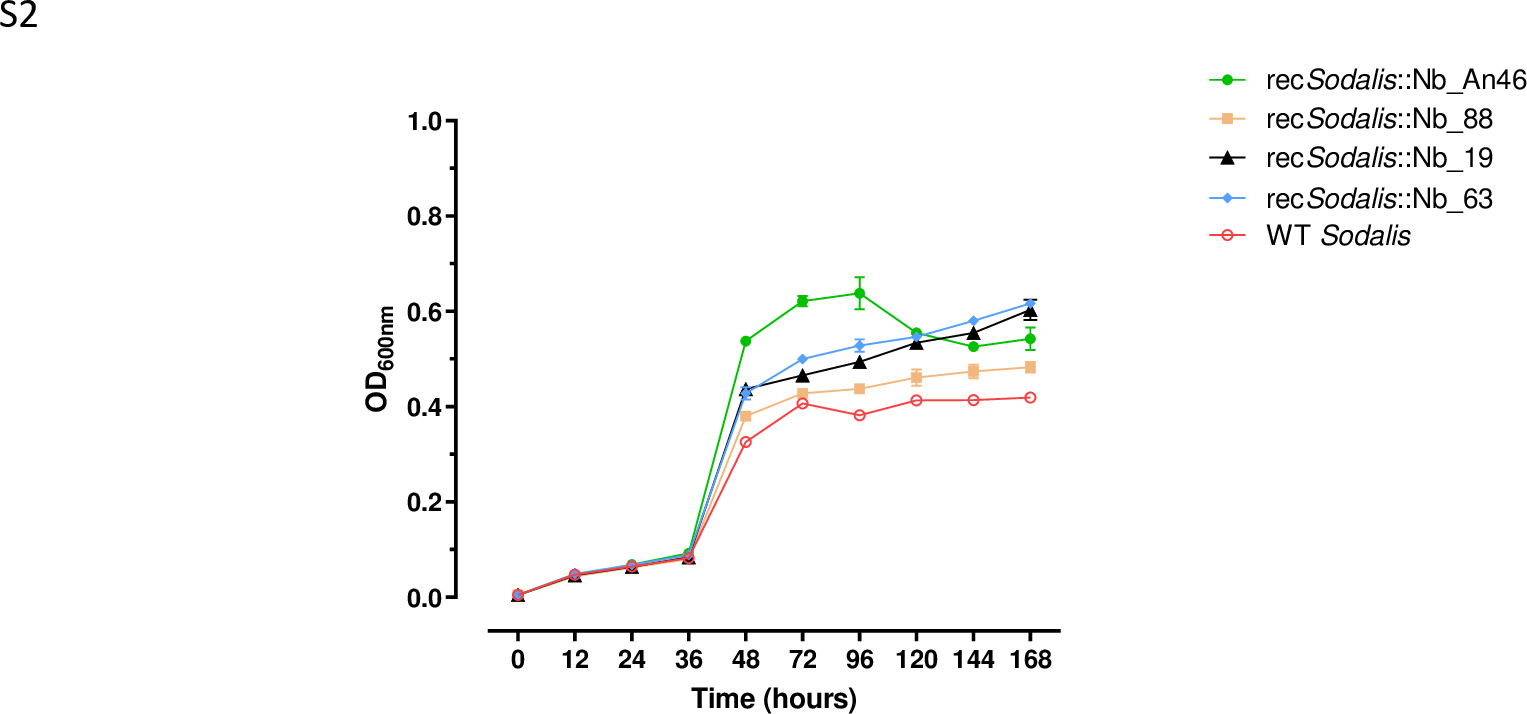

Supplement: S2 Fig — Growth curve analysis of S. glossinidius expressing recombinant Nb_An46 (green curve), Nb_88 (orange curve), Nb_19 (black curve), Nb_63 (blue curve) and WT Sodalis (red curve). The error bars show the ± SD of two biological replicates. Samples were taken every 24h except during exponential growth (0h-48h), 2 samples/24h were taken. (TIF) [file ppat.1010376.s002.tif]

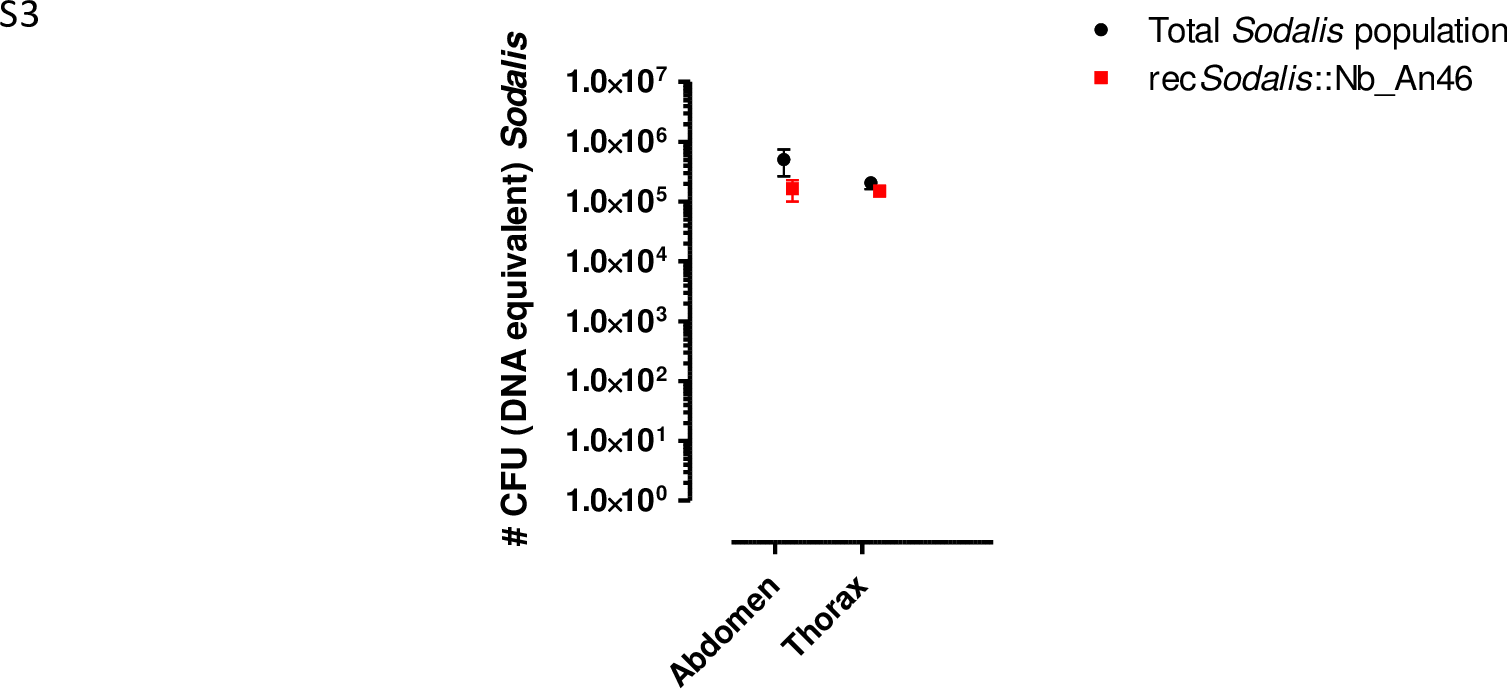

Supplement: S3 Fig — Number of recSodalis::Nb_An46 present in abdomen and thorax of teneral male flies emerged from larvae injected with 5 x 10^6^ recSodalis::Nb_An46 versus the total number of Sodalis (WT + recSodalis::Nb_An46). The number of WT and recSodalis::Nb_An46 CFUs was estimated using an already validated quantitative real time-PCR protocol (9). The bars represent the mean total Sodalis and recSodalis::Nb_An46 CFUs (+/- SD) present in abdomen and thorax of at least 5 individual flies. The number of CFUs is represented in log scale on the y-axis. (TIF) [file ppat.1010376.s003.tif]

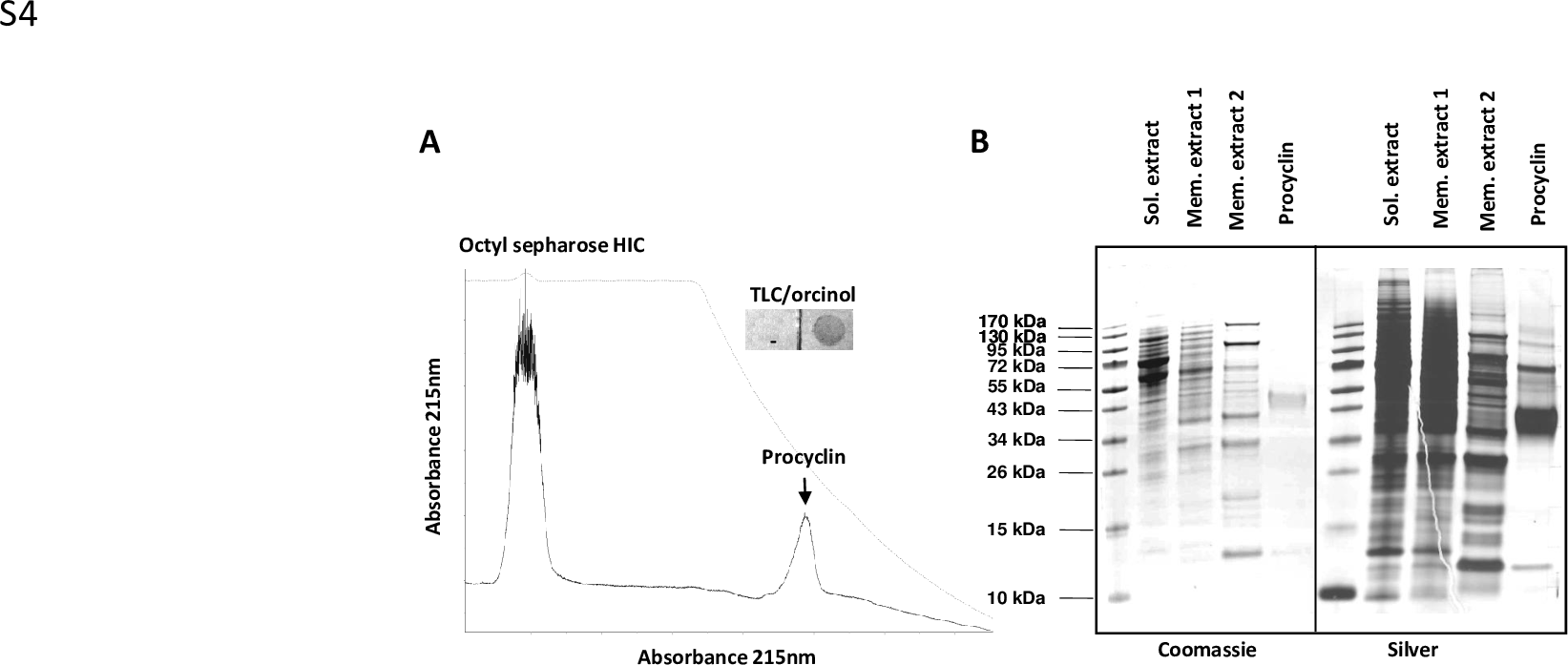

Supplement: S4 Fig — T. b. brucei EP-procyclin purification: (A) Chromatogram (OD215 nm) illustrating the procyclin purification by hydrophobic interaction chromatography on an octyl sepharose column. Eluted peak fractions were evaluated in an orcinol staining. (B) Coomassie and silver stained protein profiles of a soluble procyclic extract, two consecutive membrane extractions and purified EP-procyclin. (TIF) [file ppat.1010376.s004.tif]

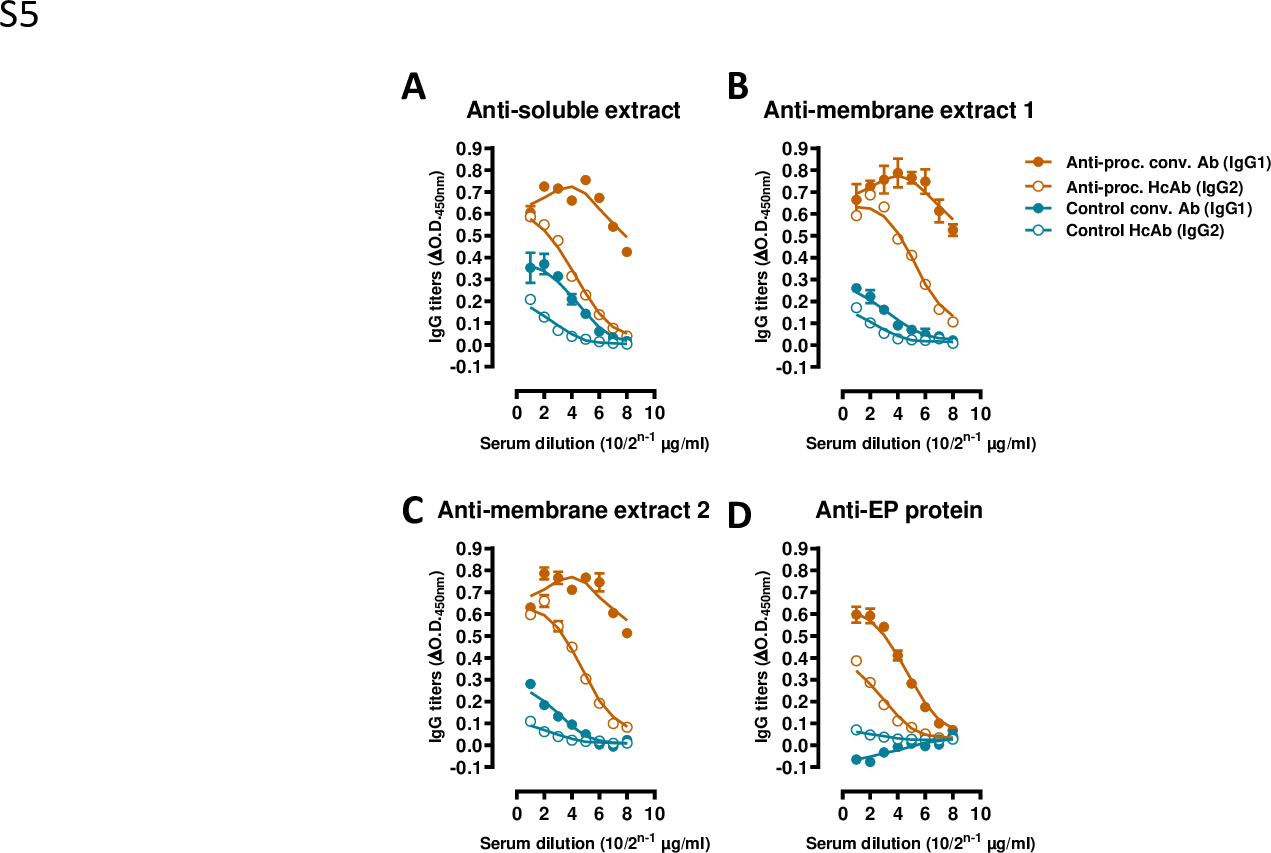

Supplement: S5 Fig — The graphs depict the reactivity of the conventional (IgG1) and heavy-chain antibody isotypes (IgG2) purified from the serum of immunized (red) and control immunized animals (blue) against (A) soluble procyclic extract, (B) procyclic membrane extract 1, (C) procyclic membrane extract 2 and (D) purified procyclin. Serial ½ serum dilutions were applied to each antigen followed by IgG detection, using an in-house rabbit anti-camel polyclonal IgG and a peroxidase-conjugated anti-rabbit IgG (Sigma). (TIF) [file ppat.1010376.s005.tif]

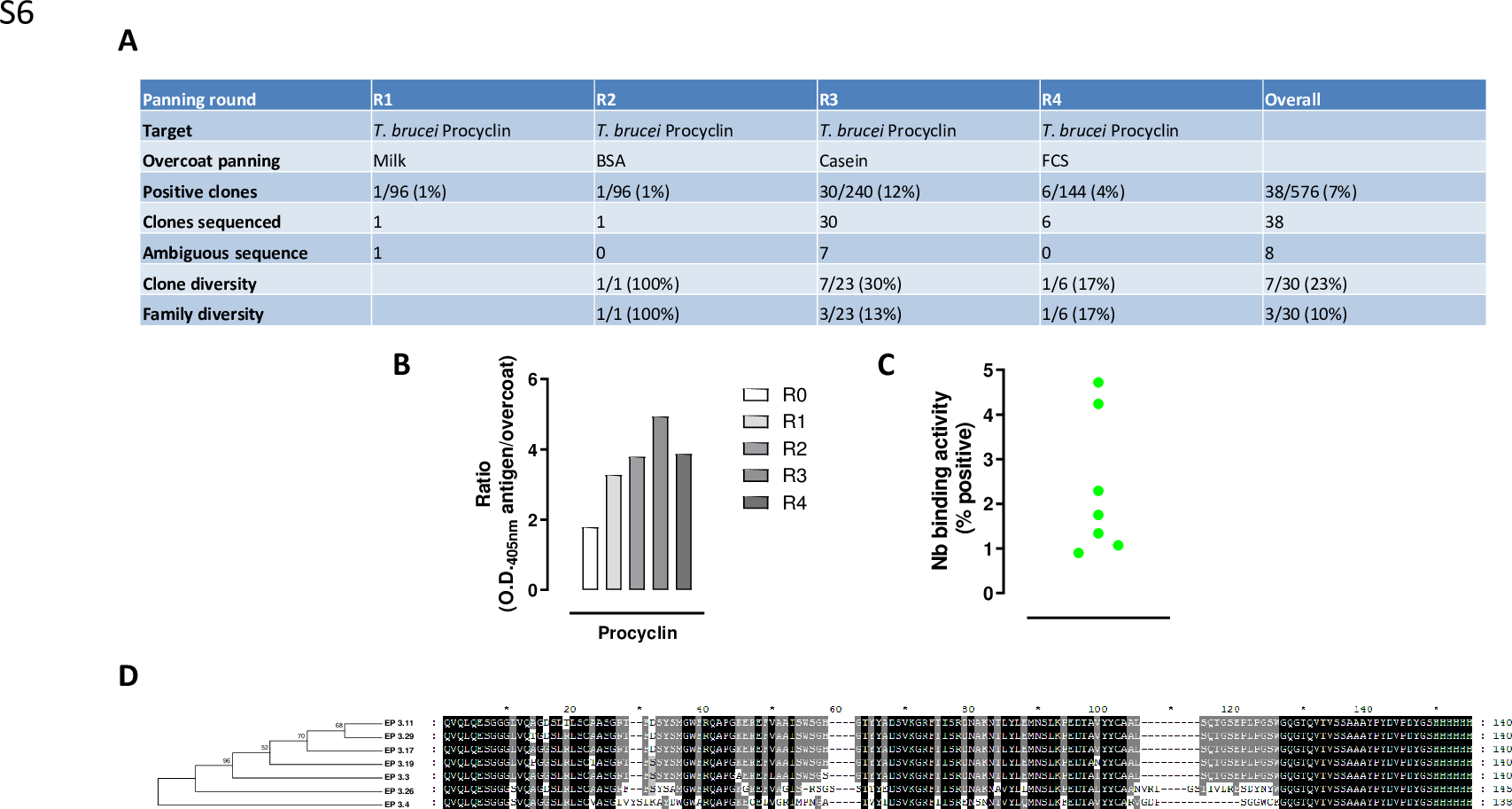

Supplement: S6 Fig — Panning of the anti-procyclic T. b. brucei surface Nb library against purified EP-procyclin: (A) Overview of the different panning rounds (Rx) with numbers of positive clones and the resulting clone and family diversity. (B) Enrichment of procyclin-specific phages throughout the different panning rounds determined by phage ELISA. (C) Flow cytometry analysis to evaluate binding activity of the individual purified Nbs (n = 7) selected onto live parasites, revealed by a one-step detection using Nb-Alexa Fluor 488 conjugates (expressed as percent positive relative to non-stained control population). (D) Alignment of the different selected Nb clones with a corresponding maximum likelihood tree. (TIF) [file ppat.1010376.s006.tif]

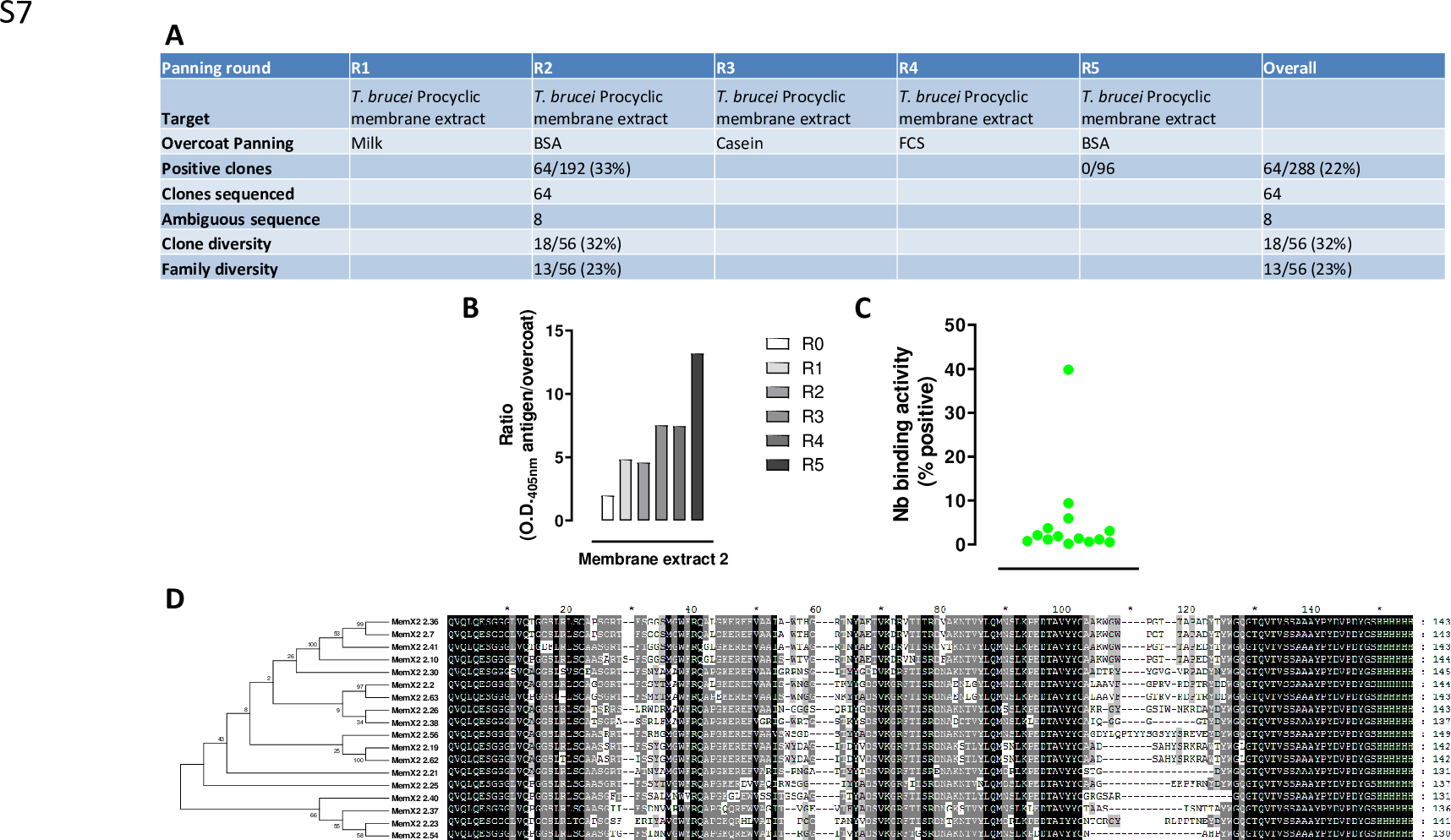

Supplement: S7 Fig — Panning of the anti-procyclic surface T. b. brucei Nb library against the second procyclic membrane extract: (A) Overview of the different panning rounds with numbers of positive clones and the resulting clone and family diversity. (B) Enrichment of specific phages reactive against the procyclic membrane components throughout the different panning rounds determined by phage ELISA. (C) Flow cytometry analysis to evaluate binding activity of the individual purified Nbs (n = 18) onto live parasites, revealed by a one-step detection using Nb-Alexa Fluor 488 conjugates (expressed as percent positive relative to non-stained control population). (D) Alignment of the different selected Nb clones with a corresponding maximum likelihood tree. (TIF) [file ppat.1010376.s007.tif]

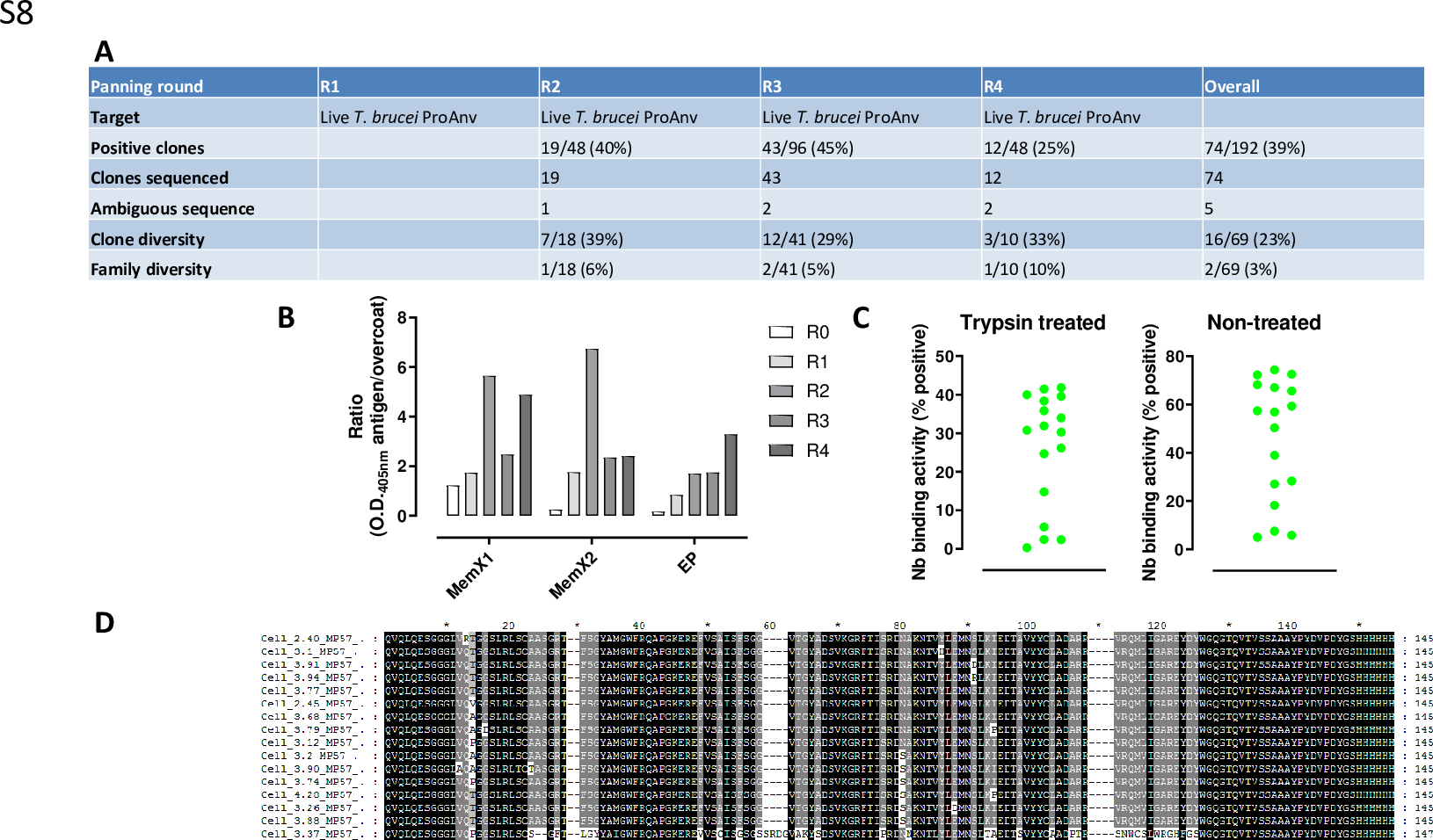

Supplement: S8 Fig — Panning of the anti-procyclic surface T. b. brucei Nb library against live procyclic trypanosomes in suspension (A) Overview of the different panning rounds with numbers of positive clones and the resulting clone and family diversity. (B) Enrichment of specific phages reactive against the different procyclic membrane extracts and EP-procyclin (EP) throughout the different panning rounds determined by phage ELISA. (C) Flow cytometry analysis to evaluate binding activity of the individual periplasmic extracts onto live procyclic trypanosomes, either (left panel) or not (right panel) pre-treated with trypsin to mimic surface antigen trimming in the tsetse midgut (expressed as percent positive relative to non-stained control population). Nbs bound to the trypanosome surface were detected using an Alexa Fluor 488 labeled anti-HA Tag antibody (1/500 dilution, Covance). (D) Alignment of the different selected Nb clones with a corresponding maximum likelihood tree (TIF) [file ppat.1010376.s008.tif]

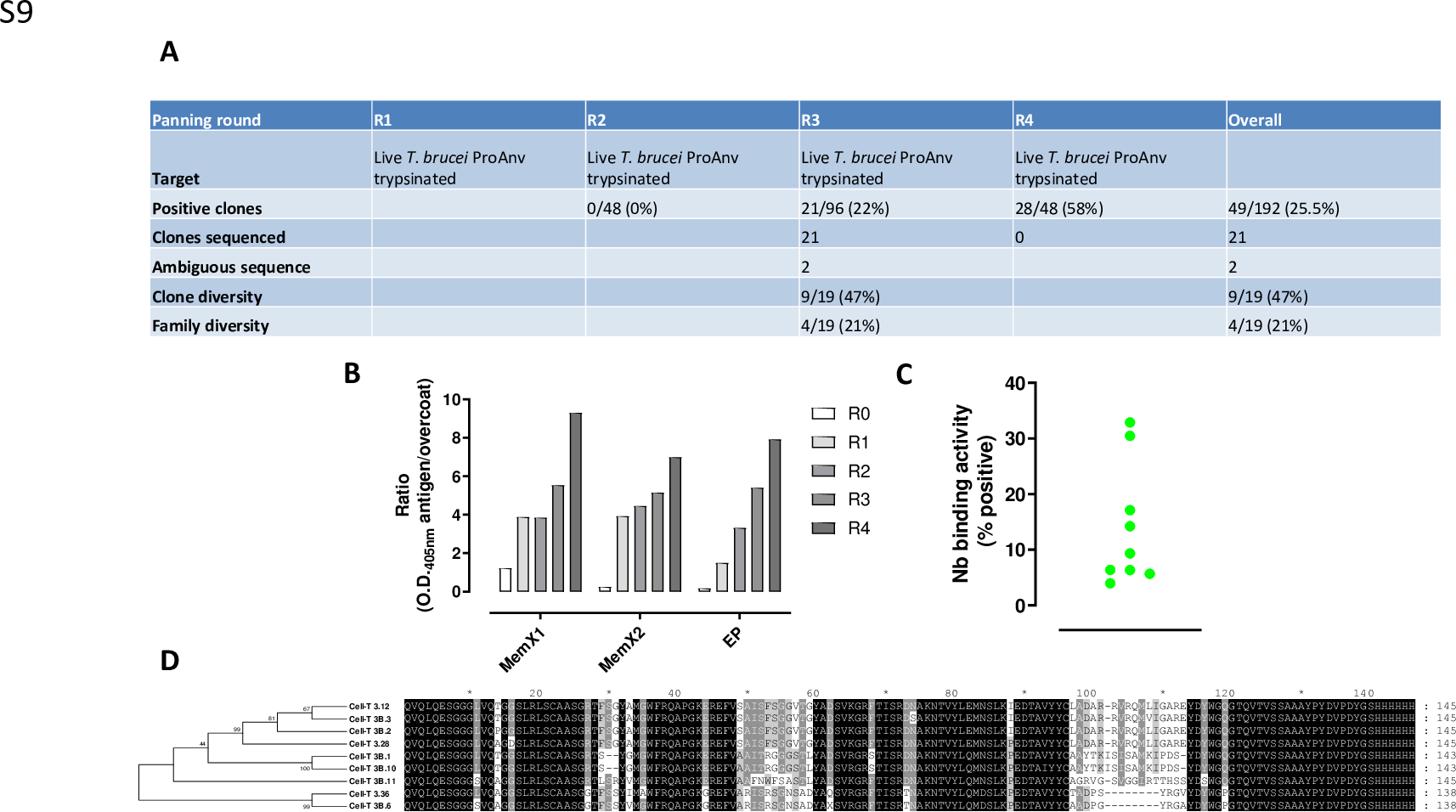

Supplement: S9 Fig — Panning of the anti-procyclic surface T. b. brucei Nb library against live, trypsinized procyclic trypanosomes in suspension (A) Overview of the different panning rounds with numbers of positive clones and the resulting clone and family diversity. (B) Enrichment of specific phages reactive against the different procyclic membrane extracts and procyclin throughout the different panning rounds determined by phage ELISA. (C) Flow cytometry analysis to evaluate binding activity of the individual periplasmic extracts onto live, trypsinated procyclic trypanosomes (expressed as percent positive relative to non-stained control population). Nbs bound to the trypanosome surface were detected using an Alexa Fluor 488 labeled anti-HA Tag antibody (1/500 dilution, Covance). (D) Alignment of the different selected Nb clones with a corresponding maximum likelihood tree. (TIF) [file ppat.1010376.s009.tif]

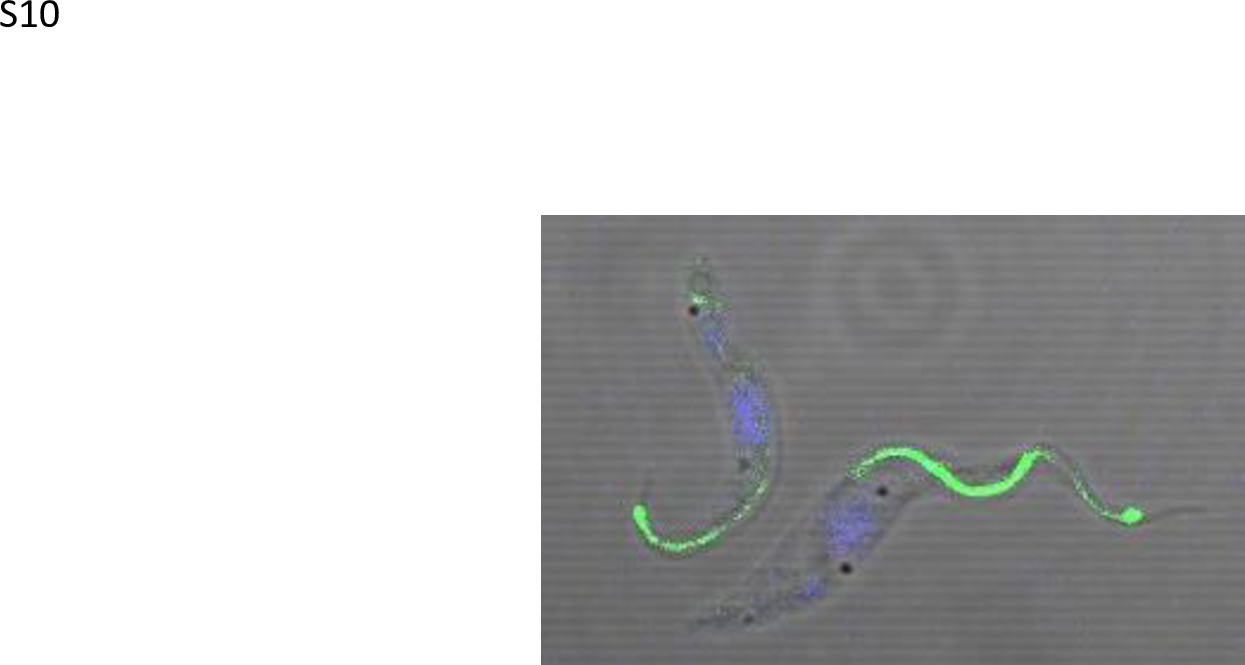

Supplement: S10 Fig — Analysis of the samples by confocal microscopy showed a staining of the flagellum. DAPI (blue) stained the nucleus and kinetoplast. (TIF) [file ppat.1010376.s010.tif]

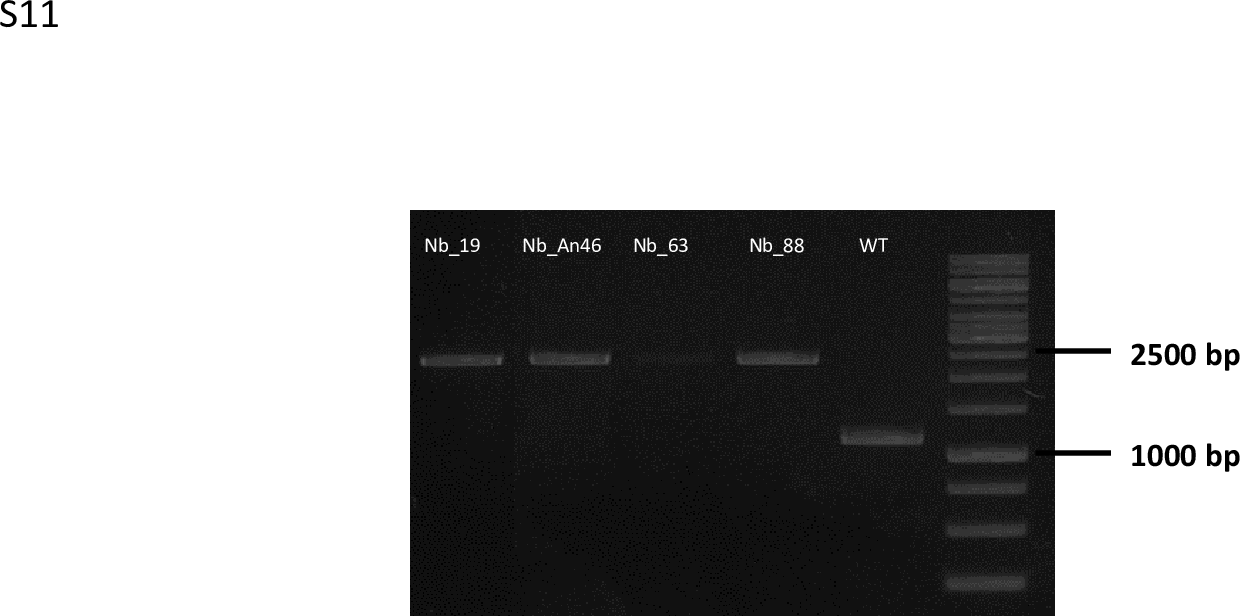

Supplement: S11 Fig — The recombinant clones all have an approximately 1265 bp higher molecular-weight band compared to the WT clone resulting from the insertion of the lac promoter:Nb cassette (approximately 825 bp) + transposon ends Tn7L and Tn7R (440 bp). (TIF) [file ppat.1010376.s011.tif]
