# Supplementary material for: Targeting the tsetse-trypanosome interplay using genetically engineered Sodalis glossinidius
Source: PLoS Pathog. 2022 Mar 10;18(3):e1010376. doi: 10.1371/journal.ppat.1010376 (PMC8939806; doi:10.1371/journal.ppat.1010376)
Supplement: Attachment — Submitted filename: Response to reviewers_De VooghtetalPPATHOGENS-D-21-02249.docx [file ppat.1010376.s012.docx]

Editorial office

PLOS PATHOGENS	Antwerp, February 8, 2022

Dear Editor,

We wish to thank you and the reviewers for the constructive comments and proposed revisions of our manuscript PPATHOGENS-D-21-0224 entitled “Targeting the tsetse-trypanosome interplay using genetically engineered *Sodalis glossinidius*”. We have addressed the questions that were raised point by point and have modified the manuscript accordingly.

This manuscript is not under consideration for publication elsewhere and all the authors agree with the submission. We thank you for considering our manuscript and look forward to your decision.

Sincerely yours,

Yours sincerely,

Linda De Vooght

Jan Van Den Abbeele

**Reviewer #1: It is an excellent, well-developed scientific work with well-defined objectives and an experimental system coordinated and consequent to them.
I have no particular remarks on the more strictly technical aspects although I would have liked some more details, at least in the discussion phase, on the r****eason for a rather limited number of samples analyzed and above all on the possible involvement of the genetic background of the insect (different strains of the vector could they offer different results?).
The main points are on the drafting of the manuscript: in the introduction many of the results are anticipated, consequently a substantial revision of the introduction could offer a better fluidity of reading and consequently an easier understanding of the technical aspects.
Similarly, many technical details are included in the results that are redundant with respect to the correct descriptions placed in the M&M section.**

- ***Reason for a rather limited number of samples analyzed****.*

The nature of *Sodalis glossinidius*-based paratransgenic experiments in tsetse does not lend itself to high-throughput testing of large numbers of samples for the following reasons: i) The female tsetse fly has a unique (but very slow) reproductive biology whereby it gives birth to a single 3rd-instar larva at about 10-day intervals throughout its reproductive life. To evaluate the effect of rec*Sodalis* on the fly’s susceptibility to trypanosome infection, the rec*Sodalis* strain needs to be microinjected into this third-instar larval stage to result in stably rec*Sodalis*-colonized tsetse flies at the freshly emerged (teneral) stage. Therefore, obtaining large samples sizes is technically highly challenging since one needs to collect and inject a high number of freshly ‘born’ larvae simultaneously at the 3^rd^ instar larval stage which only lasts for +/- 30 minutes; after that the larvae pupate and cannot be injected anymore. Of these injected larvae a small percentage (10-20%) will not survive the microinjection. Approximately 30 days after the injection, +/- 50 % of the flies that emerge from the injected larvae will be males, but only the ones that emerge within a time frame of 24h can be used for an infection experiment, otherwise the variation between the individual flies becomes too large resulting in highly variable infection rates; ii) An additional challenge is the fastidious nature of the *Sodalis glossinidius* bacterium, which has proven to be difficult to manipulate in the laboratory due to its low growth rate, high level of susceptibility to contamination, and complex nutritional requirements. For these reasons only a selection of Nbs could be assessed for the *in vivo* characterization. Notwithstanding these above stated challenges, we have reached a sufficiently high number of flies in the infection experiments to make statistically relevant conclusions.

We elaborated on these challenges in the discussion Line: 360

- ***The possible involvement of the genetic background of the insect (different strains of the vector could they offer different results?***

The genus *Glossina* consists of three species groups (*Morsitans*, *Palpalis* and *Fusca*), each of which presents differential vector competencies. Flies in the *Palpalis* subgroup are highly refractory to trypanosome infection, while those in the *Morsitans* subgroup are more susceptible. Given that *Glossina morsitans morsitans* are efficient vectors for *T. brucei brucei*, they are considered as a good experimental fly model to study trypanosome infection. We expect that in a more refractory species (like *G. fuscipes* for instance) the phenotypic impact of rec*Sodalis* on trypanosome infection could be more pronounced but this would need to be experimentally confirmed. Furthermore, it has been experimentally proven that *Sodalis* strains from two distinct host species are similar enough to interspecifically colonize tsetse flies (Weiss et al. 2006). This bypasses the need to genetically modify tsetse fly-specific *Sodalis* strains, streamlining future paratransgenesis experiments.

We have elaborated on this in the discussion part: Line 413

- ***In the introduction many of the results are anticipated, consequently a substantial revision of the introduction could offer a better fluidity of reading and consequently an easier understanding of the technical aspects****.*

We have revised the introduction and removed the paragraphs that showed redundancy with the results section.

**Reviewer #2: This manuscript describes an attempt to develop a new approach to interfere with Trypanosoma development in the tsetse fly, by engineering a fly symbiont to produce antibodies that recognize parasite surface molecules. I have a few concerns, mostly related to format.**

*•* ***The experiments are heavily biased to find nanobodies (Nbs) to procyclin, without providing the reader a rationale. It is only much later, in the Discussion, that this is addressed. Given that the majority of the readers are likely to not be Trypanosoma experts, this rationale needs to be explained up front.***

The rationale was to develop Nbs that target live trypanosomes in the tsetse midgut. As the ingested bloodstream form trypanosomes differentiate immediately into procyclic forms, we focused on these procyclic trypanosomes and more specifically on their surface coat proteins as targets since the latter are the most abundant and accessible. Therefore, the Nb library was made by immunizing an alpaca with a mixture of EP-procyclin and a procyclic trypanosome membrane extract (membrane extract 2). EP-procyclin is the most abundant surface antigen found exclusively on the procyclic forms, but we also included the membrane extract as an unbiased approach to target additional/new epitopes other than EP-procyclin on the surface of living tsetse midgut trypanosomes. Targeting intracellular proteins would theoretically also be possible but would require the Nbs to be taken up by the procyclic parasites via the endocytic machinery, of which the rate of endocytosis has been shown to be much lower compared to bloodstream form trypanosomes.

We agree with the reviewer that the rationale for our focus on surface coat proteins should be explained in the introduction and therefore we have introduced the following paragraph in the introduction (Line 139):

“ Since affinity is an important determinant of the anti-trypanosomal activity of Nbs (12), binding to an accessible, conserved epitope with sufficiently high affinity is necessary to achieve the required saturation. To meet these challenges, we generated a Nb library directed against procyclic surface proteins by immunizing an alpaca with a mixture of purified EP-procyclin and a procyclic trypanosome membrane extract of which EP-procyclin represents a highly abundant surface protein of insect-stage PF trypanosomes known to cover the whole cell uniformly expressed by both early and late procyclic forms (13). The membrane extract was included as an unbiased approach to target additional/new epitopes other than EP-procyclin.”

***• Line 194. “The second membrane extract was found to be highly enriched in membrane proteins with a lower abundance of soluble components as compared to the first membrane extraction (Suppl. Fig. 4B)”. I could not understand how this conclusion was made based on the data shown in the figure.***

After the second wash, we found that a number of protein bands (especially an abundant band below 15 kDa) that were present in the soluble fraction and also in the first membrane extract were eliminated from the second fraction. We therefore reasoned that a lower abundance of soluble components was present in the second membrane extract.

We agree with the reviewer that the sentence was a bit overstated so we changed the sentence to: “The second membrane extract was found to contain a lower abundance of soluble components as compared to the first membrane extraction (Suppl. Fig. 4B).”

• ***Lines 197–200. I could not understand Suppl. Fig. 5.***

Fig. 5 shows the results from an indirect ELISA which was used to assess the serum antibody response against the soluble extract, the two procyclic membrane extracts and the purified EP-procyclin following alpaca immunization. For this, conventional (IgG1) and heavy chain antibodies (IgG2) were purified from the plasma by protein A and protein G sepharose affinity chromatography. As controls, the same IgG fractions were also purified through the same procedure from an alpaca immunized against a non-related antigen. Immunization of the alpaca with a mixture of purified EP-procyclin and procyclic membrane extract 2 resulted in strong antibody responses against the different procyclic extracts and purified EP-procyclin, both in the conventional (IgG1) and heavy chain antibody (IgG2) isotypes.

We renamed the figure’s axes and expanded the legend to a provide more detailed description of the results depicted in Suppl. Fig 5.

Figure legend:

ELISA-based detection of antibody responses in an alpaca immunized with a mixture of purified EP-procyclin and procyclic membrane extract 2. The graphs depict the reactivity of the conventional (IgG1) and heavy-chain antibody isotypes (IgG2) purified from the serum of immunized (red) and control immunized animals (blue) against (A) soluble procyclic extract, (B) procyclic membrane extract 1, (C) procyclic membrane extract 2 and (D) purified EP-procyclin. Serial ½ serum dilutions were applied to each antigen followed by IgG detection, using an in-house rabbit anti-camel polyclonal IgG and a peroxidase-conjugated anti-rabbit IgG (Sigma).

• ***I could not understand Suppl. Figs. 6C, 7C, 8C and 9C****.*

Figures 6C, 7C, 8C and 9C depict the Flow cytometry analysis to evaluate the binding activity of the individual Nb clones onto live procyclic trypanosomes, either or not pre-treated with trypsin to mimic surface antigen trimming in the tsetse midgut. The binding activity of the Nbs is expressed as percent positive relative to a non-stained control population.

We have simplified the figures, showing only the most sensitive method of detection and adjusted the figure’s axes and expanded the legend to a provide more detailed description of the results depicted in Suppl. Fig 6C, 7C, 8C and 9C.

• ***Suppl. Figs. 7 and 8. The legends say “Panning of the anti-procyclic T. b. brucei Nb library against the second procyclic membrane extract” (Fig. 7) or “….against the live procyclic trypansomes in suspension” (Fig. 8). This lack of clarity is confusing. It does not make sense to screen a library made against a single protein (procyclin) against a complex collection of antigens (membrane extract or live parasites). It is important to be precise and give the library a name that reflects its true origins (it was made by immunizing the alpaca not with procyclin alone, but with a mixture of this protein and a membrane extract).***

Indeed, the Nb library was made by immunizing the alpaca with a mixture of purified EP-procyclin and a procyclic trypanosome membrane extract (MemX2) derived from insect-stage procyclic form trypanosomes with the aim of selecting Nbs that target the surface of midgut procyclic trypanosomes. We have now introduced the concept and the rationale of the Nb library in the introduction and changed the name of the library to “anti-procyclic *T. b. brucei* **surface** Nb library” throughout the manuscript to reflect its origin more precisely.

• ***Suppl. Figs. 7B and 8B. Define “EP”.***

In the figure legend we have defined “EP” as “EP-procyclin (EP)”

• ***Line 244. Explain the rationale for trypsinization***.

The rationale for trypsinization is explained in Line: 205, this is were the concept was first introduced.

• ***Line 253. Explain the criteria used for the selection of clones for protein production in E. coli.***

This was based on the diversity of their amino acid sequences in complementarity determining region (CDR)3. This in now included in the manuscript (Line 248).

• ***Figure 2. Expand the legend to explain how the measurements were made. While I could understand what the red lines represent, I could not figure out neither from the legend nor from the Materials & Methods how the blue lines were generated. How were control cells stained?***

We have expanded the legend but also the MM section to explain how the measurements were made.

Figure 2: Binding of individually purified anti-procyclic Nbs onto fixed procyclic *T. b. brucei*. The red histograms indicate staining with the respective anti-procyclic Nbs and detection using an Alexa Fluor® 488 labeled anti-HA tag antibody. A non-procyclic-specific Alexa488-labelled control nanobody (cAb-BCII-10) did not significantly bind to the trypanosome surface (blue histogram).

*•* ***Line 329. Reference 13 is not appropriate. This article describes about the POSSIBILITY of using viruses for paratransgenesis, but never showed that it works. I am not aware of any subsequent publications that use this virus vector for paratransgenesis.***

We agree with the reviewer and have removed reference 13 from the manuscript.

• ***Fig. 3. State the number of independent experiments that the data in panels A and B represent. For panel A, consider signaling in the figure which data are from the first experiment and which are from the second experiment.***

The presented data in Fig. 3 are representative for at least two independent experiments.

We have now indicated in panel A which data are from the first experiment and which are from the second experiment.

*•* ***Figs. 4 AND 5. 1) Indicate the number of independent experiments that the data represent; 2)indicate the number of midguts assayed; 3) indicate what the horizontal bars represent (means?, medians?, standard deviations?).***

We have modified the legends of Fig. 4 and 5 to include the following information.

1) ) Figs. 4 and 5: Presented data are representative for two independent experiments.

2) ) Figs. 4 and 5: WT *Sodalis* n= 48	rec*Sodalis*::Nb_88 n= 50	rec*Sodalis*::Nb_19 n = 42

3) Fig. 4 : Error bars represent mean with standard error of the mean (SEM) and are based on at least two independent experiments.

Fig. 5: Error bars represent mean with standard deviation (SD) and are based on at least two independent experiments.

• ***Figure 5. Please state in the legend or in the text, the median (or mean) percent transgenic bacteria for both Nb_88 and Nb_19.***

We have now included this information the main text: Line 314-317.

*•* ***For the benefit of the non-specialist, consider explaining why only male flies were used.***

Since in tsetse flies both sexes feed on blood and transmit pathogenic trypanosomes, both males and females could be used for infection experiments. However males have been found to be more efficient vectors especially at the level of maturation compared to females. This has been suggested to be attributed to the process of larviposition (intrauterine development and parturition of live offspring) which poses a strong metabolic demand on females as this process likely results in a reduction of the amounts of free nutrients available to trypanosomes which instead go towards larval production (Macleod et al., 2007). Therefore to avoid bringing in these confounding variables from the reproduction, infection experiments in tsetse are usually performed using male flies. We have included this information in the MM section:

“Only male flies were used in the experimental set-up because the susceptibility of female tsetse flies to infection is suggested to be influenced by the presence of a developing larva in the female abdomen as a result of the tsetse's viviparous reproduction mode (Macleod et al., 2007).”

Macleod ET, Darby AC, Maudlin I, Welburn SC (2007) Factors Affecting Trypanosome Maturation in Tsetse Flies. Plos One

***Overall, the experiments seem to be well executed and the findings should be of interest to the journal readers***.

**Reviewer #3: This paper describes the use of paratrangenesis approach to produce tsetse flies refractory to Trypanosoma brucei brucei procyclic developmental stage through the production of effector molecules that can target the trypanosome-tsetse fly crosstalk via the genetically modified Sodalis. The results indicate that although genetically engineered S. glossinidius expressing Nb_88 significantly compromised parasite development in the tsetse fly midgut both at the level of infection rate and parasite load, the expression of Nb_19 by S. glossinidius resulted in a significantly enhanced midgut establishment. The results are of interest as it provides the proof-of-principle for the use of paratrangenesis to modify the susceptibility of tsetse flies to Trypanosoma infection and the represent a major step forward in the development of a control strategy based on paratransgenic tsetse flies. The manuscript well written. I do have some minor comments, which I fell need to be addressed before considering this article for publication.**

**Part II – Major Issues: Key Experiments Required for Acceptance**
Please use this section to detail the key new experiments or modifications of existing experiments that should be absolutely required to validate study conclusions.

Generally, there should be no more than 3 such required experiments or major modifications for a "Major Revision" recommendation. If more than 3 experiments are necessary to validate the study conclusions, then you are encouraged to recommend "Reject".

Reviewer #1: (No Response)

Reviewer #2: (No Response)

Reviewer #3: No major issues found

**Part III – Minor Issues: Editorial and Data Presentation Modifications**
Please use this section for editorial suggestions as well as relatively minor modifications of existing data that would enhance clarity.

***Reviewer #1: As stated above, I suggest to revise mainly the introduction in which many of the results are anticipated. A substantial revision of the introduction could offer a better fluidity of reading and consequently an easier understanding of the technical aspects.
Similarly, I suggest also a revision of the result section where too many technical details are included being redundant with respect to the correct descriptions placed in the M&M section.***

We have revised the introduction and removed the paragraphs that showed redundancy with the results section. We have transferred some parts of the results section (especially technical details of the Nb library generation) to MM section*.*

***Reviewer #2: (No Response)***

***Reviewer #3: Minor comments
1- As general comments, the materials and method need revision to indicate in many sections, details on the source of the materials used i.e. kits plasmids and reagent, volume of reaction etc., to ensure the reputability of the work.***

We have thoroughly revised the MM section to include all technical details to ensure repeatability.

***2- It seems there a common typo that the time in min was indicated as (’) i.e. in lines 510, 513, 515, 540, 559, 588, 607, 609,616, 617, 618, 646, please correct.***

We have modified the manuscript: (‘) was replaced by minutes or min.

***3- In line 537: how many washes were used, please indicate the number***.

We have indicated the number of washes used.

***4- Line 545: indicate the volume or the quantity of the DNA template.***

We have now indicated the quantity of DNA template: 10–50 ng of the purified first PCR product.

***5- Line 548: indicate the source of the pMECS and pHEN4 (hamers castermans, pMECS afgeleide).***

We have modified the manuscript accordingly; pMECS (Vincke et al., 2012); pHEN4 (Arbabi Ghahroudi et al., 1997).

***6- Line 551-552: It is not clear how the digestion with Xho1 will reduce the self-ligation, please clarify.***

XhoI is indeed not correct, the correct restriction enzyme should be XbaI. The pMECS vector contains a unique Xba I site between the PstI and NotI cloning sites for VHH. Therefore, the pMECS to be used as VHH cloning vector will be cleaved by two enzymes; however, it happens that some DNA molecules are only cleaved by one enzyme. The additional digestion with XbaI (that normally cleaves in between PstI and NotI) will prevent the self-ligation of the single cleaved molecules*.*

We have corrected this in the manuscript.

***7- Line 553-554: please indicate the source of the T4 Ligase and the final volume of the reaction***.

We have modified the manuscript accordingly; T4 Ligase (Invitrogen), final volume of the reaction (20 µl).

***8- Line 555: indicate the volume of the elution buffer used after the wash with QIAquick. Indicate the devise use for the electroporation***.

We have modified the manuscript accordingly; devise used for the electroporation (using a Bio-Rad Genpulser apparatus). We did not purify the ligation mix, this was corrected.

***9- Line 556: do you mean competent cells?***

We have changed “electroporated cells” to “transformed cells” in the text.

***10- Line 556: was the incubation under shaking?***

We have now indicated that the incubation was under non-shaking conditions.

***11- Line 676-682: was the qPCR data normalized against a house keeping gene to eliminate the pitting error?***

We used an already validated quantitative PCR (qPCR) method (De Vooght et al., 2014) for the estimation of the number of WT *Sodalis*, rec*Sodalis* cells and parasites in tsetse fly tissues. For this, triplicate cultures of WT *Sodalis* and rec*Sodalis* were serially diluted (10-fold) in PBS to yield a *Sodalis* density ranging from 10^8^ CFU/ml to 10^2^ CFU/ml. DNA was extracted from each *Sodalis* dilution followed by qPCR using primers that amplify a region of the GFP gene present in recSodalis: (GFP_Fw, 5’- TGGCCAACACTTGTCACTAC-3’ and GFP_Rev, 5’- AGAAGGACCATGTGGTC -3’) and primers that target a 120-bp region of the single-copy *Sodalis glossinidius* exochitinase (Qchi) gene present in both WT and rec*Sodalis* to determine the corresponding Ct values. Standard curves were generated by plotting these C_t_ values against the corresponding log of *Sodalis* CFU/ml. This qPCR approach in combination with the *Sodalis* CFU standard curves allowed us to estimate the CFU (DNA equivalent) values present in the tsetse fly tissues of the different experimental fly series. Parasite burdens are estimated similarly using a standard curve, generated from extracted procyclic trypanosomal DNA ranging from 3.75 × 10^5^ parasites/ml to 1.95 × 10^2^ parasites/ml allowing to estimate the corresponding number of parasites present in the tsetse midgut. Used primers target the *T. b. brucei* ribosomal 18S gene: 18S_Fw (5’-TGGGGACAGTACGATGGCAGAGC-3’) and 18S_Rev (5’-TCATAGGCGGTCGGGGATAATTGCG-3’)

**12- The resolution of figure 1B, 3A, need improvement.**

We have modified the manuscript accordingly.

***13- The scientific name in the following references need to be italic (1, 3, 7, 8, 10, 11, 13, 17, 19, 20, and 23)***

We have modified the manuscript accordingly.
